# Morphogenesis and axis specification occur in parallel during optic cup and optic fissure formation, differentially modulated by BMP and Wnt

**DOI:** 10.1098/rsob.180179

**Published:** 2019-02-13

**Authors:** Priska Eckert, Max D. Knickmeyer, Lucas Schütz, Joachim Wittbrodt, Stephan Heermann

**Affiliations:** 1 Department of Molecular Embryology, Institute of Anatomy and Cell Biology, Faculty of Medicine, University of Freiburg, 79104 Freiburg, Germany; 2 Faculty of Biology, University of Freiburg, Schaenzlestrasse 1, 79104 Freiburg, Germany; 3 Centre for Organismal Studies, Heidelberg University, 69120 Heidelberg, Germany

**Keywords:** optic cup, optic fissure, morphogenesis, BMP, Wnt, coloboma

## Abstract

Optic cup morphogenesis is an intricate process. Especially, the formation of the optic fissure is not well understood. Persisting optic fissures, termed coloboma, are frequent causes for congenital blindness. Even though the defective fusion of the fissure margins is the most acknowledged reason for coloboma, highly variable morphologies of coloboma phenotypes argue for a diverse set of underlying pathomechanisms. Here, we investigate optic fissure morphogenesis in zebrafish to identify potential morphogenetic defects resulting in coloboma. We show that the formation of the optic fissure depends on tissue flow movements, integrated into the bilateral distal epithelial flow forming the optic cup. On the temporal side, the distal flow translates into a ventral perpendicular flow, shaping the temporal fissure margin. On the nasal side, however, the distal flow is complemented by tissue derived from the optic stalk, shaping the nasal fissure margin. Notably, a distinct population of TGFβ-signalling positive cells is translocated from the optic stalk into both fissure margins. Furthermore, we show that induced BMP signalling as well as Wnt-signalling inhibition result in morphogenetic defects of the optic fissure. Our data also indicate that morphogenesis is crucial for a proper positioning of pre-specified dorsal–ventral optic cup domains.

## 1. Introduction

Eye morphogenesis in vertebrates is a complex process during which initially optic vesicles evaginate from the eye field, located within the late prosencephalon [1–3]. Subsequently, these optic vesicles are transformed into bi-layered optic cups [4,5]. During transformation, a physiological but transient cleft emerges, at their ventral pole, termed the optic fissure [4,5]. The fissure is important during a specific period of development, in which it is used by cells of the periocular mesenchyme (POM) and by embryonic vasculature to enter the eye. Yet it is essential that the optic fissure is closed as development proceeds. A persisting optic fissure, termed coloboma, is a frequent cause for congenital blindness [6]. A plethora of genes have been linked to coloboma formation [7], resulting in a coloboma gene network [8,9]. This network is growing and consists among others of components of various signalling pathways, such as Wnt [10,11], FGF [12,13], RA [14,15], Hippo [16], Shh [17] BMP [18] and TGFβ [19]. It is noteworthy that the morphology of coloboma phenotypes resulting from alterations within these signalling pathways is highly variable. Alterations in some result in a subtle coloboma phenotype [12,13,19], potentially involving the alignment or even the fusion of the optic fissure margins. However, alterations in the other result in a vast, extended coloboma phenotype [11,16]. It becomes more and more clear that eye morphogenesis *per se* is a dynamic process. During optic cup morphogenesis, next to a bending of the neuroepithelium driven by basal constriction [20,21], dynamic tissue rearrangements have been described [18,22–25]. It became evident that lens-averted domains are secondarily integrated into the forming optic cup via a bilateral tissue flow/migration over the distal rim [18]. We hypothesize that at least some of the mentioned vast coloboma phenotypes are the result of morphogenetic defects during optic cup formation, as previously demonstrated by the precocious arrest of the ‘neuroretinal flow’ [18]. We propose that morphogenetic defects affecting the optic cup also affect the formation of the optic fissure specifically. To date, it is largely unclear how most of the mentioned signalling pathways are affecting optic cup morphogenesis in general, or optic fissure morphogenesis in particular. Even though it is becoming more and more evident that optic cup morphogenesis is a dynamic process, the morphogenesis of the optic fissure itself is not well understood. The current understanding of optic fissure morphogenesis is not yet taking into account the overall cell and tissue dynamics during eye morphogenesis. Currently, it is still largely believed that the optic fissure is generated by a bending of the nasal and temporal cup domains [4].

Here, we addressed the morphogenesis of the optic fissure and the assembly of the optic fissure margins using zebrafish (*Danio rerio*). We further analysed how this is affected by BMP and Wnt signalling. We find that under normal conditions, the bilateral neuroretinal flow [18] at the temporal side directly translates into a ventral perpendicular tissue flow. This is forming the temporal optic fissure margin. At the nasal side, we also observed a ventral flow, however, largely originating from the optic stalk, especially in the proximal domain of the optic cup. Notably, we find that subsequently a distinct cell population, in which TGFβ signalling was activated, is translocating from the optic stalk into both fissure margins. We furthermore show that BMP4 induction and Wnt inhibition both result in a morphogenetic defect of the optic fissure. BMP4 induction was next to this also resulting in a dorsalization of the ventral optic cup, in line with previous findings [26,27]. Notably, Wnt-signalling inhibition was not altering the dorsal–ventral axis specification. However, the affected morphogenesis due to Wnt-signalling inhibition did result in a mispositioning of distinct parts of optic cup domains. Thus, our data indicate that morphogenesis and axis specification are occurring in parallel during optic cup formation and need to be addressed together. Otherwise, a misinterpretation of the formation of misshaped domains would be a likely consequence. Overall, we provide evidence that tissue dynamics are essential for optic fissure morphogenesis and that these are largely affected by BMP and Wnt-signalling. We propose that morphogenetic defects are also the reason for other ‘vast coloboma phenotypes'.

## 2. Results

### 2.1. Overall tissue dynamics driving optic cup and optic fissure morphogenesis

**Figure 1.**
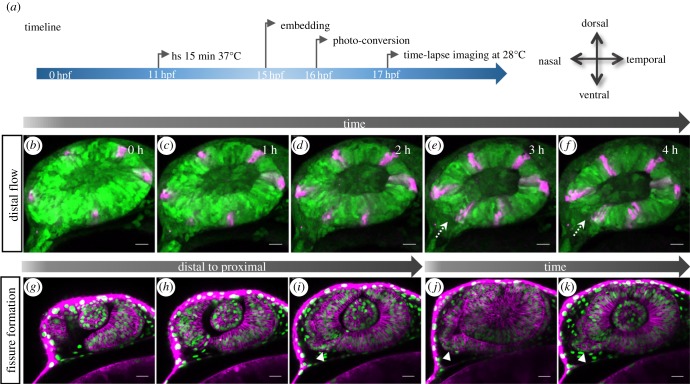
Transformation of the optic vesicle into the optic cup. (*a*) Timeline of experimental procedure (for *b*–*f*) and orientation of the eye. (*b*–*f*) Distal flow and fissure generation. (*b*) Photo-converted domains (magenta) in the lens-averted layer move over the distal rims into the lens-facing domain (*c*–*f*). During this process, the fissure is induced (dotted arrow). (*g*–*i*) The optic fissure is generated from distal to proximal. (*i*–*k*) The optic stalk (arrowhead) is connected to the nasal lens-averted domain of the optic vesicle, imaging starts (*g*–*j*) at 19 hpf; scale bar, 25 µm.

To address the tissue dynamics during optic cup formation, we employed *in vivo* time-lapse imaging of *tg(hsp70:kaede*) embryos. Kaede is a photo-convertible fluorescent protein. Kaede expression was induced after a heat shock applied at 11 hpf for 15 min. Specific domains of the developing eye were photo-converted from green to red fluorescence (figure 1*a*). Following the photo-converted cells over time, a gastrulation-like rearrangement of lens-averted cells into the lens-facing domain can be appreciated (figure 1*b–f*; electronic supplementary material, movie S1). Note the marked flat cells (magenta, figure 1*b*), located in the lens-averted domain, which over time reach the lens-facing domain of the optic cup and elongate (figure 1*c*–*f*). The flow over the distal rims, which has been analysed previously [18], is nicely visible also from a lateral perspective. Importantly, this flow is bilateral, which allows the nasal and temporal domains to extend distally. This can be interpreted as the onset of optic fissure formation, demonstrated in a nuclear- and membrane-labelled optic cup (figure 1*g*–*i*).

Notably, the optic vesicle is connected to the optic stalk (figure 1*i*, arrow). In a transition zone, the stalk is connected to the lens-averted domain of the optic cup in a triangular manner (figure 1*j*, arrow). This connection can be found on the nasal side and the morphology of this transition zone is changed over time (figure 1*j,k*).

### 2.2. Establishment of the temporal optic fissure margin

**Figure 2.**
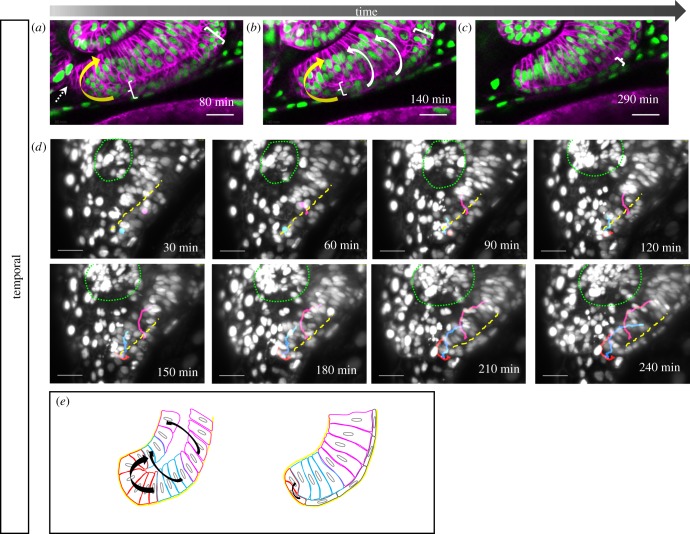
Development of the temporal fissure margin. (*a*–*e*) Close up of the developing temporal ventral optic cup domain, (*a*–*c*) labelled with *tg(bact:H2BGFP)* and *tg(bact:lyntdTomato)*, cells from the lens-averted layer of the optic cup (*a*, bracket) are flowing over the distal rims (*a*, *b* white arrows) and in a perpendicular direction (*a*, *b*, yellow arrow) over the ventral margin into the lens-facing layer (6 fish in 4 experiments). (*c*, bracket) Cells in the lens-averted layer (RPE domain) flatten and obtain RPE cell shape. Lateral view, nasal to the left; scale bar, 25 µm. (*d*) Mosaic nuclear labelling (H2BGFP mRNA injection), maximum projection of 70 optical sections corresponding to 70 µm (*z*-spacing was 1 µm). Tracking of single cells (from one eye) moving over the distal (magenta and blue, *n* = 2 for both, respectively) and the ventral distal (red, *n* = 4) rim (dashed yellow line), respectively, into the lens-facing layer of the prospective neuroretina. Lens marked with green dotted line. Lateral view, nasal to the left; scale bar, 25 µm. (*e*) Scheme of temporal fissure margin development. Cells move over the distal (magenta), the distal ventral (blue) rims and via a ventral perpendicular flow (red) over the ventral rim (black arrows) from the lens averted into the lens-facing layer.

After this global analysis of tissue dynamics, we focused on the region of the forming optic fissure. At first, we addressed the temporal fissure margin. To visualize the cell dynamics, we made use of a transgenic line with ubiquitous nuclear and membrane labelling (*tg(bact2:H2BGFP, bact2:lyntdTomato)*). In the lateral perspective, the prospective neuroretinal tissue, located within the lens-averted layer of the temporal ventral domain, is nicely visible (figure 2*a*,*b*, between white brackets). In the lateral aspect, these prospective neuroretinal cells are moved over the distal rim into the lens-facing domain of the optic cup (figure 2*a*,*b*, white arrows).

Furthermore, it can be appreciated that in the ventral aspect, prospective neuroretinal cells move over a ventral rim via a perpendicular flow into the temporal lens-facing domain of the optic cup (figure 2*a*,*b*, yellow arrow; electronic supplementary material, movie S2). This ventral rim corresponds to the temporal fissure margin. After optic cup morphogenesis is complete, the lens-averted layer is made up of flattened cells, the prospective RPE cells (figure 2*c*, bracket).

To facilitate a better individual cell tracking, we reduced the density of labelled nuclei by injecting RNA coding for H2BGFP into one cell at the 4- to 8-cell staged embryo in the context of a ubiquitous membrane labelling, achieved by injections of RNA coding for lyntdTomato into the zygote. In addition, we increased the resolution in *z* during time-lapse imaging by performing an exemplary single-plane illumination imaging (SPIM, lightsheet) experiment. Three-dimensional volume analysis over time allowed us to follow individual single cells, artificially labelled with coloured dots, from the lens-averted domain on their way into the optic cup (figure 2*d*; electronic supplementary material, movie S3). The distal rim (figure 2*d*, dashed line) and the ventral rim can be appreciated in the max projection of the three-dimensional volume. Individual cells were followed over the distal and distal–ventral rim (magenta and blue) and over the ventral rim (red). This indicates that cells enter the optic cup via a ventral perpendicular flow, which is a direct continuation of the distal flow [18] on the temporal side. The findings are summed up in a scheme (figure 2*e*).

### 2.3. Establishment of the nasal optic fissure margin

**Figure 3.**
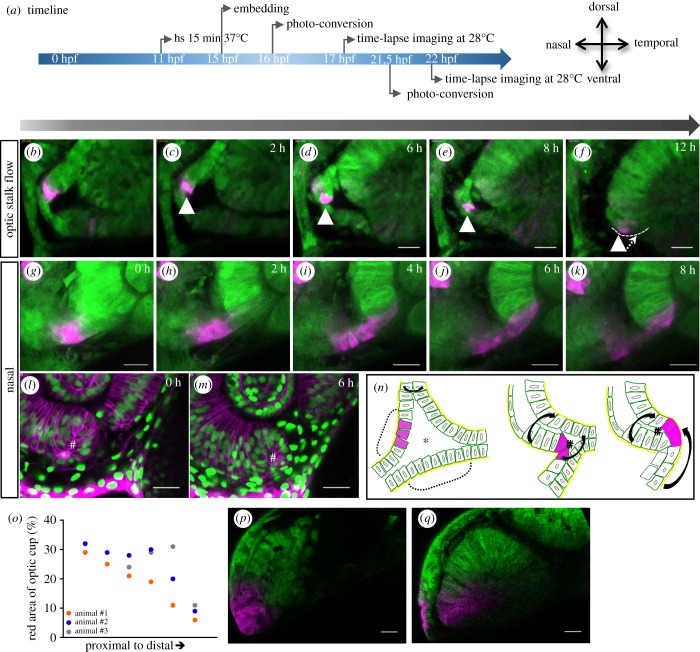
Development of the nasal fissure margin. (*a*) Timeline of experimental procedure and orientation of the eye (timeline for *b*–*f* displayed on top of the arrow, for *g*–*k* below). (*b*–*f* and *g*–*k*) Close ups of the developing nasal ventral optic cup labelled with *tg(HSP70::kaede)*, photo-converted cells (magenta) derived from the optic stalk translocate from the optic stalk into the nasal fissure margin (12 fish in 6 experiments; not all of them are kaede experiments). Lateral view, nasal to the left, scale bar: 25 µm. (*l*–*m*) In distal domains, a turbulence or vortex of cells can be appreciated (hash), in this domain, two flow movements collide, one over the distal rim, one from the optic stalk. Cells remaining in the RPE domain flatten and thus obtain RPE cell shape (labelled by *tg(bact:H2BGFP)* and *tg(bact:lyntdTomato)*). (*n*) Scheme of nasal fissure margin development. In proximal regions of the early optic cup, the optic stalk consists of two layers (dashed brackets), which are connected to the lens-averted domains of the optic cup (see also figure 1*g*) and border the optic ventricle (asterisk). Over time, the upper layer is moving into the optic fissure margin. The distal and optic stalk flow movements are indicated by black arrows. The twist of the optic stalk could easily be driven by the distal flow on the nasal side which is flowing over the optic stalk. Eventually, stalk-derived cells are integrated via the nasal fissure margin. (*o*) Optic stalk contribution to the ventral neuroretina (stalks of three animals were converted and the red area was measured separately). (*p*) Optic vesicle from animal #2 before fissure development. Parts of the optic stalk were converted. (*q*) Optic cup from animal #2 after fissure development. Photo-converted cells moved into the ventral area of the optic cup. Scale bar, 25 μm.

We have now established that on the temporal side, the ventral and the distal flow are directly linked (figure 2). On the nasal side, however, the optic stalk is connected to the optic vesicle, probably influencing the morphogenesis of the fissure margin in concert with the distal flow. To address the stalk contribution to fissure margin morphogenesis, we made use of the *tg(hsp70:kaede*) line again. We first photo-converted in the upper transition zone (figure 3*b*) to address the onset of stalk contribution. It can be appreciated nicely that the upper domain of the transition zone is moving into the fissure margin over time (figure 3*b*–*f*), changing the overall morphology of the transition zone from a triangular shape to a flat shape (figure 3*b*–*f*; electronic supplementary material, movie S4), what we termed ‘twist’ of the optic stalk. In order to visualize the further extent of the optic stalk contribution, we specifically labelled a domain within the optic stalk by photo-conversion of Kaede using embryos of the *tg(hsp70:kaede)* line. The photo-converted domain was then followed in a three-dimensional volume over time (figure 3*g*–*k*). It can be appreciated nicely that also this distinctly labelled domain is over time integrated from the optic stalk into the nasal optic fissure margin (figure 3*g*–*k*; electronic supplementary material, movie S5). These data are in line with previous observations [24,28]. Notably, in the ventral nasal domain, in which the distal flow and the stalk flow merge, a turbulence can be observed, which we termed ‘vortex’ (figure 3*l*,*m*, #; electronic supplementary material, movie S6). The findings are summed up in a diagram (figure 3*n*). To provide further quantitative data for the stalk contribution, we performed another set of experiments in which we photo-converted parts of the stalk domain early and analysed the contribution of this domain in the optic cup later (figure 3*o*–*q*; electronic supplementary material, figure S3 supplement). We found a stalk contribution ranging from 10 to 30%, being higher in the proximal regions of the optic cup and decreasing distally (figure 3*o*; electronic supplementary material, figure S3 supplement).

### 2.4. Additional TGFβ-signalling-positive cells enter from the optic stalk

**Figure 4.**
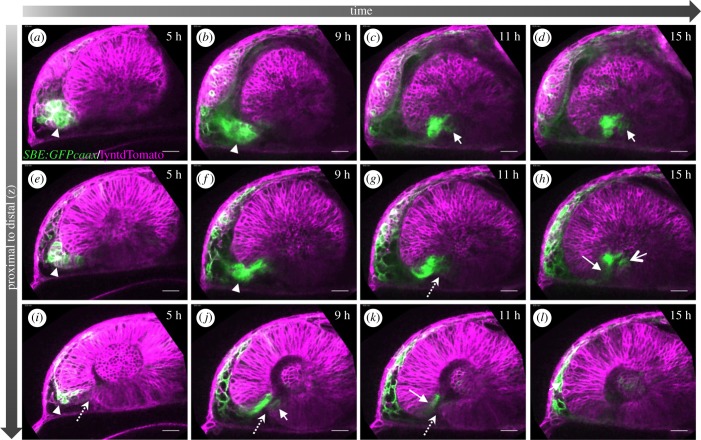
TGFβ-signalling-positive cells are secondarily added to the optic fissure margins. (*a*–*l*) Four-dimensional dataset of the developing optic cup, TGFβ reporter (green), cell membranes (lyntdTomato, magenta). Presented are three different optical planes (top to bottom) over time (left to right). TGFβ reporter activity in the optic stalk (*a*,*e*,*i*, arrowhead). TGFβ-signalling-positive cells move from the optic stalk into the optic cup (*c*–*k*, arrow) (8 fish in 4 experiments; 3 fish in 2 experiments). The optic stalk is predominantly connected to the nasal optic cup (*a*,*b*,*e*,*f*). At the end of the flow, TGFβ-signalling-positive cells populate the most proximal domain of the optic fissure (*c*,*d*,*f*,*g*) (3 fish in 2 experiments). From here, these cells also populate the temporal fissure margin (*c*,*d*,*f*,*g*,*h*,*j*). The optic fissure is marked with a dotted arrow. Lateral view, nasal to the left; scale bar, 25 µm.

We recently found that TGFβ signalling is essential for optic fissure fusion [19]. In this context, we observed active TGFβ signalling within the optic fissure margins using an *in vivo* TGFβ-signalling reporter in zebrafish [19]. Here, we addressed whether the TGFβ-signalling domain is extending into the margins by signalling activation within the margin cells, or by secondarily translocation of cells, in which TGFβ signalling was already activated, into the margins. To this end, we performed *in vivo* time-lapse imaging of embryos of the *tg(SBE:GFPcaax)*, injected with RNA coding for lyntdTomato. We identified a domain in the forebrain and optic stalk, in which TGFβ signalling was activated (figure 4*a*,*e*,*i*, arrowhead). We then followed cells of this domain over time. We found that the active TGFβ-signalling domain was extending through the optic stalk (figure 4; electronic supplementary material, movies S7–S9). We identified TGFβ-signalling-positive cells which were translocated from this domain into the nasal fissure margin secondarily (figure 4; electronic supplementary material, movies S7 and S8). Notably, although the optic stalk was found to be connected predominantly to the nasal fissure margin, we observed TGFβ-positive cells crossing to the temporal fissure margin via the prospective optic nerve head (figure 4*c*,*d*,*f*–*h*; electronic supplementary material, movies S7 and S8). In distal fissure domains, the reporter signal is lost over time, due to either a continuous flow of these cells into other domains or due to a reduction in signalling activity over time (figure 4*j*,*k*).

### 2.5. BMP signalling induction and inhibition of Wnt secretion affect optic fissure morphogenesis

At this point, we had a framework for optic fissure formation in the context of optic cup morphogenesis at hand. Next, we wanted to address whether this morphogenetic process is hampered in conditions which are known to result in coloboma with a vast cleft, and if so, how. To this end, we made use of two models. On the one hand, we made use of an artificial expression of bmp4. On the other hand, we made use of an inhibition of porcupine, a factor which is crucial for the secretion of active Wnt ligands [29,30].

#### 2.5.1. BMP induction

**Figure 5.**
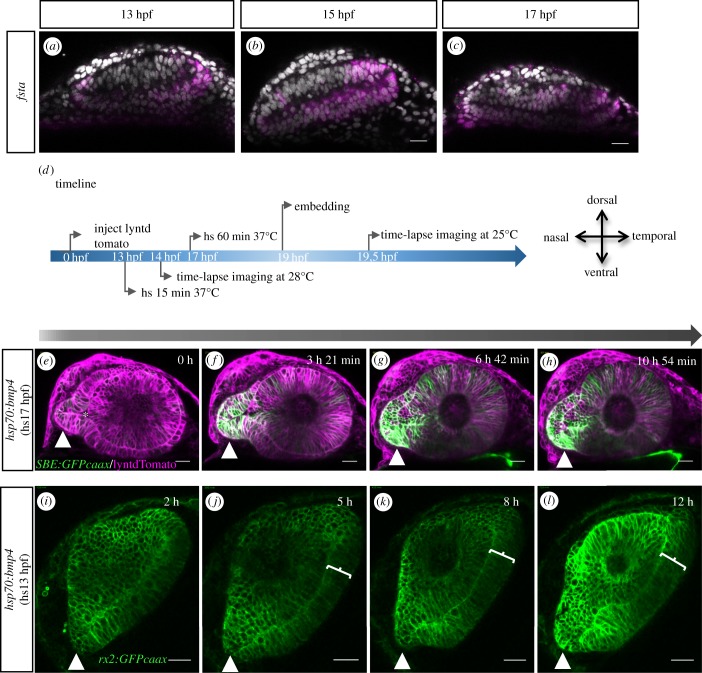
Induced expression of bmp4 hampers optic fissure formation. *In situ* hybridizations for *fsta* (*a*–*c*) for 13, 15 and 17 hpf in WT embryos. *fsta* is expressed, from temporal to the ventral transition zone to the optic stalk. (*d*) Timeline of the experimental procedure and orientation of the eye, heat shocks (hs) performed at 17 hpf are displayed on top of the arrow, hs performed at 13 hpf below the arrow. (*e*–*h*) Lateral view of optic cup development in the *tg(hsp70:bmp4)* background, visualized by lyntdTomato (mRNA), *tg(SBE:GFPcaax)*. *bmp4* induced at 17 hpf hampers proximal optic fissure morphogenesis (10 fish in 1 experiment). The optic stalk is in continuation to the lens-averted domains of the developing optic cup (*e*, arrowhead). Asterisk marks the optic ventricle. In temporal and dorsal regions, the lens-averted layer is being integrated into the optic cup (*f–h*). Cells from the lens-averted layer of the optic cup are not properly integrated into the lens-facing domain. The connection of the optic stalk to the lens-averted domain is maintained and the optic fissure is not formed in the proximal domain. The TGFβ signalling activity can be seen in the optic cup, in the absence of orderly tissue dynamics. See figure 4 as control. (*i*–*l*) Lateral view of optic cup development in the *tg(hsp70:bmp4)* background, visualized by *tg(rx2:GFPcaax)*. *bmp4* induced at 13 hpf results in an absence of the optic fissure (4 fish in 1 experiment). The optic stalk is misshaped (arrowhead). On the temporal side, a persisting lens-averted domain is visible (brackets *j*–*l*). Scale bar, 25 μm. See electronic supplementary material, figure 5 supplement 1 O–X as control.

First, we addressed the effect of induced BMP signalling. It was previously shown that the bilateral distal flow could be precociously arrested by induced BMP expression [18], the likely reason for this being an oversaturation of the BMP antagonist follistatin a (*fsta*), which was found expressed in the nasal and temporal domain of the forming optic cup [18]. We now addressed the expression of *fsta* also in the forming ventral aspect of the optic cup. Notably, we find it expressed in the transition zone from the stalk to the lens-averted domain (figure 5*a*–*c*). We then crossed fish from *tg(hsp70:bmp4, cmlc2:GFP)* with fish from *tg(SBE::GFPcaax)*, both described previously [19], to produce double transgenic embryos. Heat shock inductions of bmp4 were performed at 17 hpf. Subsequently, the embryos were subjected to *in vivo* time-lapse imaging (figure 5*d*). bmp4 inductions starting at 17 hpf resulted in overt morphogenetic defects of the optic cup (figure 5*e*–*h*). The optic stalk and the TGFβ-signalling active domain within were found enlarged (figure 5*e*–*h*), compared to controls (figure 4*e*–*h*) and the connection of the optic stalk to the optic cup was found broadened. A clear symmetry breaking connection of the stalk to a future nasal domain including a twist of the optic stalk could not be identified. Importantly, the optic stalk was found stuck and not integrated into the optic cup (electronic supplementary material, movie S10). Nevertheless, under these conditions without proper tissue dynamics, TGFβ-signalling active domains were later found within the optic cup but also within lens-averted domains (figure 5*f*–*h*). Notably, TGFβ-signalling activity within the optic cup can be observed during retinal differentiation (not shown). The perpendicular flow on the temporal domain was also largely absent (electronic supplementary material, movie S10). Notably, however, the distal flow in the ventral domains was not abrogated, explaining that in distal domains, an optic fissure was forming (electronic supplementary material, S1A–D and movie S11). This was at odds with findings from embryos in which bmp4 was driven from the *rx2 cis*-regulatory element, in which especially the distal flow on the temporal side was arrested [18] (electronic supplementary material, figure S5 supplement 1, E–N, see O–X as control and movies S13 (proximal), S14 (distal), see S15 (proximal) and S16 (distal) as control). Notably, in this scenario, the optic stalk is also stuck and the perpendicular flow on the temporal side is largely absent (electronic supplementary material, figure S5 supplement 1, E–I and J–M). Notably, to a certain extent, a pathologically formed fissure can be found in distal domains [18] (electronic supplementary material, figure 5 supplement 1, N). We next performed heat shock inductions of *bmp4* at 13 hpf. Subsequently, the embryos were subjected to *in vivo* time-lapse imaging (figure 5, timeline). Bmp4 inductions starting at 13 hpf resulted in overt and even more pronounced morphogenetic defects of the optic cup (figure 5*i*–*l*; electronic supplementary material, figure S5 supplement 3, showing the overall morphology post-heatshock at 13 hpf and 17 hpf). Large ectopic putative neuroretinal domains can be appreciated (figure 5*i*–*l*; electronic supplementary material, movie S12). Importantly, also the stalk-derived flow as well as the perpendicular flow on the temporal side were found absent resulting in an absence of the optic fissure (figure 5*i*–*l*; electronic supplementary material, figure S5 supplement 2, A–D). Accordingly, the optic fissure is not only an important entry route for the POM and blood vessels, which enter through the fissure, but also for prospective neuroretinal tissue which is integrated into the ventral optic cup via the optic fissure margins. Thus, it is comprehensible that large domains of the ventral optic cup must be missing. We therefore addressed the ocular phenotype resulting from a *bmp4* induction via heat shock 13 hpf at a later time point. Notably, at 40 hpf, the lens is protruding ventrally, explainable by missing ventral optic cup domains (electronic supplementary material, figure S5 supplement 2, E–H). So, even though no optic fissure was forming, at later developmental stages, a ventral gap is visible, not corresponding to the normal optic fissure. Since the overall flow movements were arrested drastically, the shape of the optic cup including the ‘abnormal ventral fissure’ must be resulting predominantly from a bending of the cup, probably caused by ojoplano-mediated basal constriction [20,21].

#### 2.5.2. Wnt inhibition

**Figure 6.**
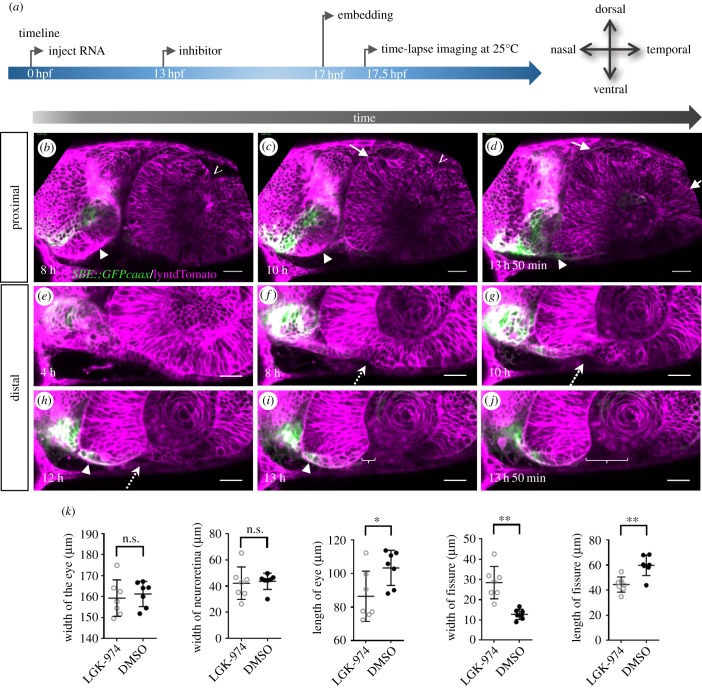
Wnt-signalling inhibition affects optic cup morphogenesis and prevents TGFβ-signalling positive cells from entering the ventral part of the optic cup. (*a*) Timeline of the experimental procedure, and orientation of the eye. (*b*–*j*) Four-dimensional dataset of a developing optic cup. TGFβ reporter (green); cell membranes (lyntdTomato, magenta). One optical section in a proximal (*b*–*d*) and one in a distal region (*e*–*j*) over time (left to right). TGFβ-signalling-positive cells are located in the misshaped optic stalk/forebrain (arrowhead). Few TGFβ-signalling-positive cells reach the nasal ventral part of the developing optic cup (*h*,*i*, arrowhead). The dorsal fissure (*b*–*c*, marked with v) seems to close over time. Ectopic domains of the presumptive neuroretina can be seen in the lens-averted dorsal domain (*c*–*d*, arrows). Even though TGFβ-positive cells do not move into the eye, a nasal fissure margin is visible. On the temporal side, the ventral perpendicular flow seems corrupted, affecting the formation of the temporal fissure margin. The distal flow in the ventral domains, both nasal and temporal, seems unaffected resulting in an optic fissure being visible in distal domains (*i,j* brackets). A dotted arrow indicates where the fissure will open (5 fish in 1 experiment, 3 TGFB reporter, 2 WT). Scale bar, 25 µm. (*k*) Quantification of distinct morphological parameters of LGK-974-treated embryos versus DMSO-treated embryos. *p < 0.05; **p < 0.005.

We next addressed the role of Wnt signalling during optic cup and fissure formation. In a mouse knockout, the loss of porcupine has been shown to result in a coloboma with a vast cleft and ectopic neuroretina [11]. We inhibited porcupine with a small compound inhibitor (LGK-974, Hycultec GmbH, Beutelsbach) mimicking the coloboma phenotype observed in mouse. We hypothesized that the vast coloboma could well result from a hampered optic fissure morphogenesis, even though the interpretation of the ocular phenotype found in the porcupine KO mouse was a different one [11]. We made use of *in vivo* time-lapse imaging using embryos with the TGFβ-signalling reporter transgene as a stalk marker, in which we injected with lyntdTomato RNA at the zygotic stage. The embryos were transferred to fish-medium containing the compound inhibitor LGK-974, an inhibitor for porcupine, at 13 hpf. To test the efficacy of the inhibitor, we performed a dose–response analysis for the inhibitor (electronic supplementary material, figure S6 supplement 2) and also made use of an established Wnt reporter in zebrafish, *tg(7xTCF-Xla.Siam:GFP)* (electronic supplementary material, figure S6 supplement). A reduced Wnt-signalling activity can be appreciated nicely. Strikingly, and according to our hypothesis, the inhibition of porcupine was also resulting in a defect of the flow movements during optic cup morphogenesis (figure 6; electronic supplementary material, movies S17 and S18). This can nicely be appreciated in the dorsal domain of the optic cup, where ectopic domains of the prospective future neuroretina can be observed on the nasal and the temporal side (figure 6*b*,*c*, arrows). However, even more strikingly, the domain of TGFβ-signalling positive cells was found largely stuck within the optic stalk (figure 6*c*,*d*, arrowhead). In addition, this also negatively affected the ventral perpendicular flow on the temporal side (electronic supplementary material, movie S18). Moreover, we quantified distinct parameters (figure 6*k*). While the width of the optic cup was unchanged, its length was reduced. We also noted an increased width of the optic fissure, while its length was reduced. Overall, our data demonstrate that Wnt ligands are important for optic cup and optic fissure morphogenesis. The data furthermore indicate that the coloboma phenotype resulting from a hampered Wnt signalling is a secondary, morphogenetic coloboma.

### 2.6. Dorsal ventral axis specification during optic cup formation and the role of BMP and WNT-signalling

**Figure 7.**
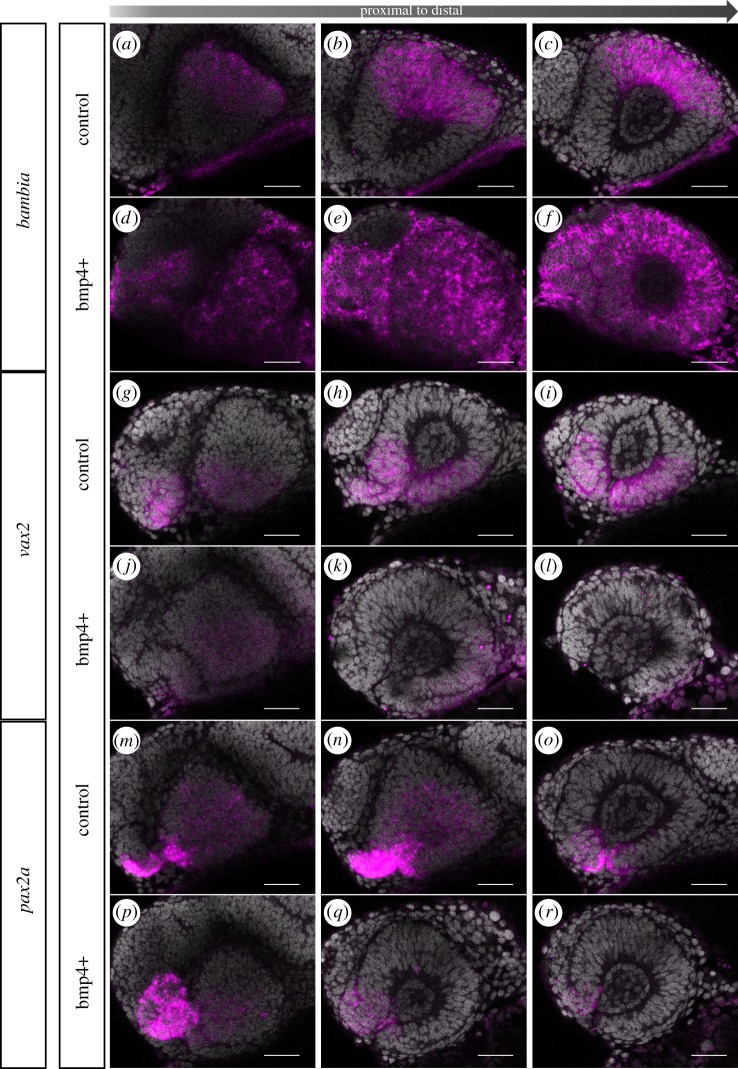
Induced bmp4 expression affects moprphogenesis and axis specification. *In situ* hybridizations for *bambia* (*a*–*f*), *vax2* (*g*–*l*) and *pax2a* (*m*–*r*) in *tg(hsp70:bmp4)* (*d*–*f*, *j*–*l*, *p*–*r*) and control embryos (*a*–*c*, *g*–*i*, *m*–*o*) at 24 hpf after heat shock at 17 hpf. Note the extended *bambia* expression domain within the entire optic cup resulting from bmp4 induction, compared to the dorsal expression domain in the control. *Vax2* expression is reduced after bmp4 induction compared to the control. *Bmp4* induction also results in a reduced *pax2a* expression domain within the optic cup, while the *pax2a* expression domain in the optic stalk is enlarged. Lateral view, nasal to the left; scale bar, 25 µm. For each condition, eight embryos were used; four to five of them were imaged.

A hampered optic fissure morphogenesis was observed resulting from both induced *bmp4* expression and porcupine inhibition. However, next to affecting morphogenetic tissue dynamics, BMP and Wnt-signalling both could affect the dorsal ventral axis within the optic cup [31]. We thus next addressed if *bmp4*-induced signalling and Wnt-signalling inhibition was also affecting the identity of cells within the optic cup. We addressed the expression of several markers, known to be expressed in distinct domains of the optic cup, namely *bambi* [18,32] in the dorsal optic cup, *vax2* [32,33] in the ventral optic cup and *pax2a* [33] in the optic stalk and ventral most optic cup. *bmp4* induction, performed at 17 hpf, resulted in a dramatic increase in the *bambia* expression domain. While *bambia* can be found expressed in the dorsal domain of the optic cup in controls (figure 7*a*–*c*), *bmp4* induction increased the expression domain to the entire optic cup, the optic stalk and large domains of the forebrain (figure 7*d*–*f*). The expression of *vax2*, which in controls can be found in the ventral domain of the optic cup and optic stalk (figure 7*g*–*i*), was largely absent after *bmp4* induction (figure 7*j*–*l*). Noteworthy, however, was a remaining expression of *vax2* in the lens-averted domain on the temporal side (figure 7*j*). This domain is corresponding to the domain which was stuck during morphogenesis and was not correctly integrated into the lens-facing side of the optic cup (figure 7*j*). The induction of bmp4 furthermore resulted in a reduced *pax2a* expression domain within the optic cup (figure 7, *m–o* controls, *p–n* *bmp4* over-expression). The *pax2a* expression domain in the optic stalk, however, was found enlarged (figure 7*p*).

**Figure 8.**
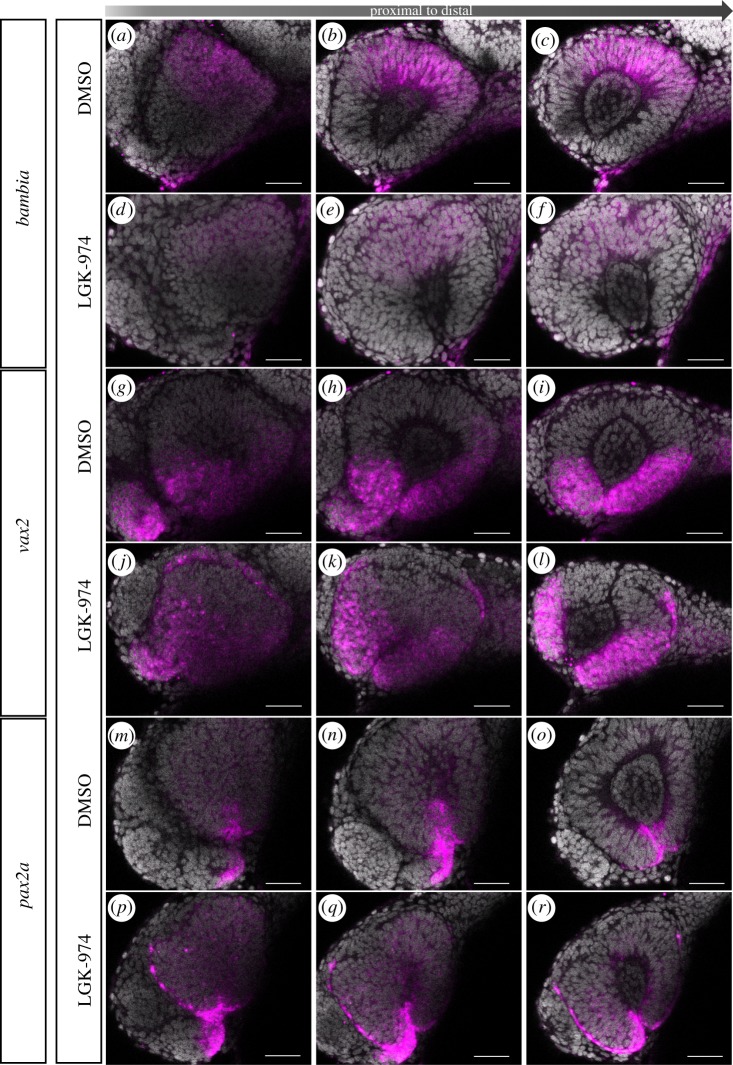
Inhibition of Wnt-signalling affects morphogenesis. *In situ* hybridizations for *bambia* (*a*–*f*), *vax2* (*g*–*l*) and *pax2a* (*m*–*r*) on porcupine inhibitor (LGK-974)-treated embryos (*d*–*f*, *j*–*l*, *p*–*r*) and DMSO control embryos (*a*–*c*, *g*–*i*, *m*–*o*) at 24 hpf. Inhibitor treatment started at 13 hpf. Wnt-signalling inhibition is not affecting the expression domain of *bambia* but reducing expression intensity. Wnt-signalling inhibition results in ectopic expression of *vax2*, which can be found in the lens-averted layer of the optic cup. Wnt-signalling inhibition also results in a reduced *pax2a* expression domain within the optic cup, while the *pax2a* expression domain in the optic stalk is enlarged and extending into the nasal lens-averted domain. Lateral view, nasal to the left; scale bar, 25 µm. For each condition, eight embryos were used; four to five of them were imaged.

**Figure 9.**
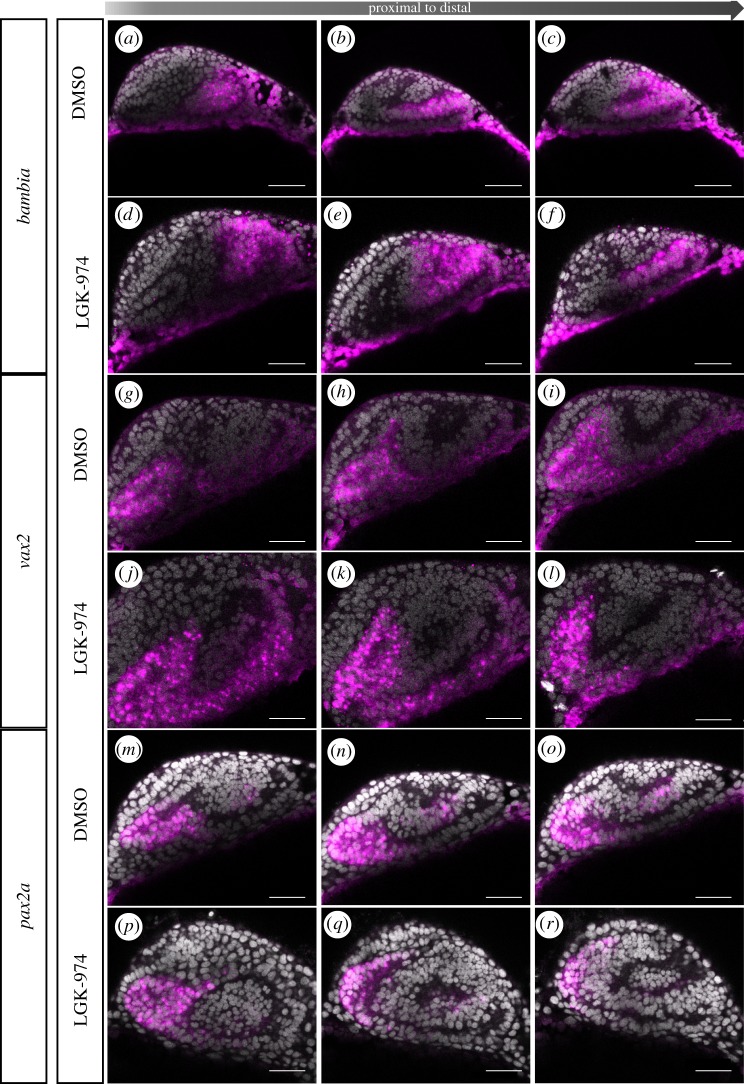
Inhibition of Wnt-signalling does not affect dorsal ventral axis specification. *In situ* hybridizations for *bambia* (*a*–*f*), *vax2* (*g*–*l*) and *pax2a* (*m*–*r*) on LGK-974-treated embryos (*d*–*f*, *j*–*l*, *p*–*r*) and DMSO control embryos (*a*–*c*, *g*–*i*, *m*–*o*) at 17 hpf. Inhibitor treatment started at 13 hpf. No change of the expression pattern was found for *bambia* and *vax2. Pax2a*, however, was found expressed ectopic within the nasal lens-averted layer resulting from Wnt-signalling inhibition. Lateral view, nasal to the left; scale bar, 25 µm. For each condition, eight embryos were used; four to five of them were imaged.

The inhibition of porcupine resulted in a reduced expression intensity of *bambia,* though the expression domain seemed largely unaffected compared to controls (figure 8*a*–*c* controls, *d*–*f* treatment). Notably, we found *bambia* expressed to a similar extent at 17 hpf in controls and porcupine-inhibited embryos (figure 9*a*–*f*), suggesting an onset of repression in between 17 hpf and 24 hpf. *Vax2* was found expressed in the ventral optic cup and optic stalk also after porcupine inhibition (figure 8*j*–*l*), in domains where it can also be found in controls (figure 8*g*–*i*). Importantly, *vax2* could also be found in lens-averted domains of the optic cup (figure 8*j*–*l*). These domains corresponded to the domains which failed to be integrated into the lens-facing domain of the optic cup correctly during morphogenesis. This is indicating that the domain of *vax2*, often used as a ventral marker for the optic cup, is largely originating from the lens-averted part of the early optic cup. We further analysed this by addressing the *vax2* expression domain at 17 hpf (figure 9*g*–*l*). It can be appreciated nicely that in controls, but also in porcupine-inhibited embryos, the vax2-positive domains can be found in the lens-averted domain of the early optic cup (figure 9*g*–*l*). The domain of *pax2a*, expressed in the ventralmost optic cup and the optic stalk in controls, was found mildly reduced after inhibition of porcupine (figure 8*m*–*r*). Notably, after porcupine inhibition, *pax2a* expressing cells could be found in the lens-averted domain on the nasal side (figure 8*p*–*r*). An extension of the *pax2a* domain from the optic stalk into the nasal lens-averted domain resulting from porcupine inhibition could be seen already at 17 hpf (figure 9*p*–*r*). See figure 9*m*–*o* as control.

#### 2.6.1. Summary, discussion and conclusion

**Figure 10.**
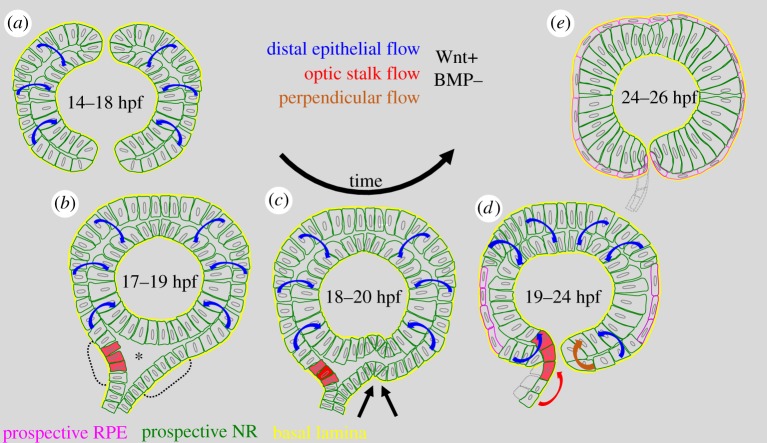
Summary of the findings for optic fissure morphogenesis. (*a*) Onset of fissure formation by a bilateral distal flow, (*b*) establishment of the triangular transition zone, (*c*) formation of an indentation in the future optic fissure region, (*d*) integration of the lens-averted domain but also of the upper transition zone into the optic fissure (red cells) in a ‘twist’ movement set-up of proper fissure margins (*e*).

Even though the role of tissue dynamics is more and more appreciated for optic cup formation [18,22–25], only very little was so far known about optic fissure morphogenesis and whether or not this also is affected by tissue dynamics. A still prevailing view suggests a mere ventral bending of the nasal and temporal domains to drive fissure formation [4]. We show, however, with *in vivo* time-lapse analyses of zebrafish embryos that tissue dynamics play a major role for optic fissure formation (see figure 10 for a summary of our findings). Based on cell tracking, we find the ‘bilateral neuroretinal flow’ over the distal rim [18] in direct continuation with a ventral perpendicular flow on the temporal side. On the nasal side, however, a tissue flow derived from the optic stalk is shaping the fissure margin, especially in the proximal domain. A certain contribution of stalk-derived cells during optic cup development was also found in *Xenopus* [28] and a similar finding was described as a late evagination in zebrafish [24]. We further uncovered a cellular vortex in the region of the nasal margin, probably resulting from converging streams of cells from the distal flow [18] and the stalk flow (this study) during which the optic stalk undergoes a twist movement. The surface ectoderm including the forming lens and the forming optic cup are suggested to interdepend due to the exchange of inductive signals [5]. Prospective neuroretinal cells, which are only secondarily integrated into the lens-facing domain of the optic cup over the distal rims, could therefore be shielded from such inductive signals, a concept potentially important for the establishment of the stem cell containing ciliary marginal zone, recently discussed [18]. Here, we show that the optic fissure margins serve as ventral rims, over which, next to the distal rims, cells are secondarily integrated into the optic cup. These cells will also be shielded from inductive signals before they enter the lens-facing domain of the optic cup. This affects the population of cells derived from the lens-averted domain of the optic vesicle but also the stalk-derived cells. Notably, we identified a translocation of a specific set of cells, which are moved from the optic stalk into the optic cup. In these cells, TGFβ signalling was activated. Even though we found the optic stalk mainly connected to the nasal fissure margin, especially in the distal aspect, the TGFβ-signalling-positive cells moved through the nasal fissure and via the presumptive optic nerve head also to the temporal fissure margin. Notably, TGFβ signalling was shown to be important for optic fissure fusion [19], a process following the orderly formation of the optic fissure margins. The translocation of the TGFβ-signalling active cells nicely shows that the secondarily integrated cells might not just be potentially shielded from inductive signals from the surface ectoderm but also might be activated or primed in proximal domains before they are sent to their destination inside the optic cup. It is also mentionable that this population is sent to both fissure margins, meaning that it is not respecting the distinct nasal and temporal domains [5,34]. The finding of a bilateral flow *per se* suggests a static dorsal and ventral pole within the forming optic cup [18]. The dorsal pole can be nicely appreciated, because it is causing the emerging ‘dorsal fissure’ [35]. The region of the ventral pole must be corresponding to the prospective optic nerve head, marking the proximal end of the optic fissure. The flow of tissue entering from the optic stalk could be a contributing factor to the proximal initiation of a split flow. Overall, such detailed knowledge about optic fissure morphogenesis is crucial to understand the origin of diverse coloboma phenotypes. Even though a defective fusion of the optic fissure margins may be the most appreciated cause for coloboma, a fusion defect is very unlikely to be the reason for coloboma in which the cleft is so big that the margins cannot touch. Such coloboma are more likely to be ‘indirect’ and rather result from optic fissure formation defects [36].

We addressed two coloboma models presenting a vast cleft as a phenotype [11,18]. We used *bmp4* induction [18,19] and small compound-mediated inhibition of porcupine. The latter is mimicking the phenotype observed in porcupine KO mice [11]. Porcupine is crucial for the secretion of active Wnt ligands [29,30]. Mutation of β-catenin resulted in a similar coloboma phenotype [10]. This is suggesting that the vast coloboma phenotypes observed in β-catenin mutants and in porcupine mutants are resulting from a hampered canonical Wnt signalling.

In both our inducible coloboma models, we found a drastic effect on the ventral invagination (summed up in figure 10). The flow derived from the optic stalk was arrested, resulting in a failure of the proximal optic fissure to form. This suggests that the optic stalk is indeed contributing to the initiation of optic fissure formation. Since *bmp4* induction is also affecting the distal flow in the ventral domains, it is understandable that the phenotype is ultimately resulting in an almost complete absence of the optic fissure. Although both coloboma models share a ventral flow defect, they showed also marked differences in other aspects. While the inhibition of porcupine resulted in a strong morphogenetic defect affecting mostly the tissue dynamics, heat shock-induced induction of *bmp4* affected the cells also in other ways. Mentionable is the ectopic activation of TGFβ signalling found during *in vivo* time-lapse imaging as well as the ectopic induction of *bambia* expression, extending through the entire optic cup and the almost completely missing *vax2* expression, which could only faintly be seen in the lens-averted domain. These effects can probably be interpreted as a shift within the dorsal ventral axis of the optic cup [26,27,32] in line with a dorsalization phenotype. The remaining, though faint, expression domain of *vax2* in the lens-averted domain, nicely visible on the temporal ectopic domain, however, underlines the failure of tissue dynamics. Our expression domain analyses of the porcupine-inhibited embryos are also indicating a morphogenetic defect. Our data clearly show that the expression domain of *vax2* is following the tissue flow. In control embryos, analysed at 17 hpf, *vax2* was found expressed largely in lens-averted domains and the optic stalk. Only later, at 24 hpf, could the classical ventral domain be seen. The inhibition of porcupine, blocking the morphogenetic tissue movements, however, resulted in the *vax2* domain largely being stuck in the lens-averted domain of the optic cup. These findings show the importance of combining time-lapse imaging analyses with expression analyses. If the tissue dynamics would have been unknown or ignored, the altered localization of *vax2* expressing cells could easily have been mistaken for a trans-differentiation of RPE precursors. The relatively normal sized, though mislocalized, *vax2* expression domain indicates a normal ventral identity of the cells making up the domain. However, the expression intensity of *bambia* in the dorsal domain was found reduced. The reason for this likely is a hampered maintenance of the dorsal cell identity found in the absence of Wnt-signalling in line with previous findings [31]. The domain of *pax2a* was found slightly reduced in the optic cup and mislocalized to the lens-averted domain and stalk. An extension from the optic stalk into this domain was already apparent at 17 hpf.

Overall, we provide a solid framework of data for optic fissure morphogenesis. This framework is the basis for the analyses of coloboma having in common a vast cleft, which are unlikely resulting from a mere defect of optic fissure fusion. As a proof of concept, we applied our framework and analysed two known vast coloboma phenotypes. It is very likely that this framework will be applicable also for many other colobomas with a vast and extended cleft. Furthermore, the concept of a defective morphogenetic process as a basis for vast cleft phenotypes will probably also be useful for other tissues (e.g. palate and neural tube).

## 3. Material and methods

### 3.1. Zebrafish

*Husbandry*. Zebrafish (*Danio rerio*) were kept in closed stocks in accordance with local animal welfare law. The fish facility is under supervision of the local representative of the animal welfare agency. Fish were maintained in a constant recirculating system at 28°C on a 12 L : 12 D cycle. Age of zebrafish embryos was determined according to Kimmel *et al.* [37].

*Transgenic zebrafish. TGFβ reporter**:** (tg(SBE:GFPcaax)* were generated previously, as described by Knickmeyer *et al*. [19], *rx2 reporter: tg(Ola.rx2:eGFP-caax)* were described and used previously [18]. *Ubiquitous nuclear and membrane reporter:* The double transgenic line containing, *tg(actb2:H2BGFP) tg(actb2:lyntdTomato)* was created in the laboratory of Joachim Wittbrodt and kept in a AB/Casper background. *Kaede reporter: tg(hsp70:kaede;cmlc2:eGFP*) was created in the laboratory of Jochen Wittbrodt and kept in a AB/WIK background*.* The two lines were kindly provided by the laboratory of Jochen Wittbrodt. *Wnt reporter:* The *tg(7xTCF-Xla.Siam:GFP)* transgenic line was kindly provided by Mathias Carl. *HS-bmp4: tg(hsp70:bmp4; cmlc2:eGFP)* were described as used previously [19]. *Rx2-driven bmp4 overexpression: tg(rx2:bmp4; cmlc2:eGFP)* was described as used previously [18].

*Transient labelling of zebrafish.* Where indicated, H2BGFP (nuclear localized GFP) (50 ng µl^−1^), lyntdTomato (75–150 ng µl^−1^) was injected into 1- to 8-cell staged zebrafish embryos enabling four-dimensional imaging of mosaic or ubiquitous labelled zebrafish.

### 3.2. Heat shock

Heat shocks for the *tg(hsp70:kaede; cmlc2:GFP)* and *tg(hsp70:bmp4; cmlc2:GFP)* lines were applied in a heating block at 37°C for different times. Early heat shocks up to 13 hpf were applied for 15 min, later they were applied for 1 h.

For *tg(hsp70:bmp4, cmlc2:eGFP)* embryos, heat shocked wild-type siblings from the same clutch of eggs were used as controls.

### 3.3. Inhibitor treatment

Porcupine inhibition: LGK-974 (Hycultec GmbH Beutelsbach) was solved in DMSO (10 mM). Embryos were treated with 20 µM LGK-974 from 13 hpf on until end of imaging. For the dose–response curve, embryos were treated with 1, 10, 20 and 50 µM of inhibitor. Control embryos were treated with an equal amount of DMSO without inhibitor.

### 3.4. Whole-mount *in situ* hybridization

Whole-mount *in situ* hybridization (WMISH) was performed according to Quiring *et al.* [38] and Heermann *et al.* [18]. WMISHs for confocal imaging were stained with FastRed Naphthol (Sigma-Aldrich). The nuclei were stained with DAPI (4 μg ml^−1^). For each *in situ* 8–10 embryos were stained, 4–6 were confocally imaged.

### 3.5. Microscopy

Whole-mount in situs were imaged with a Leica TCS SP8 confocal microscope with a *z*-spacing of 3 µm.

### 3.6. Time-lapse imaging

Time-lapse imaging was performed on inverted Leica TCS SP8 set-ups with two internal hybrid detectors, and a 40× long distance objective (water immersion), using Immersol (Carl Zeiss) as immersion medium. For time-lapse imaging, embryos at appropriate stages were embedded in 1% low melting agarose in glass bottom dishes (MatTek, Ashland, MA, USA) and covered with zebrafish medium, including tricaine for anaesthesia. Confocal stacks were taken every 10 min with a resolution of 1024 × 1024 and 3 µm *z*-spacing. The left and right eyes were used and oriented to fit the standard lateral view.

### 3.7. Processing of time-lapse imaging data

The stacks were evaluated with FIJI software [39]. For denoising of the movies, the PureDenoise plugin [40] was used with four cycle-spins and three frames multiframe.

### 3.8. Single plane illumination (lightsheet) imaging

Time-lapse SPIM imaging was performed with a Leica TCS SP8 set-up upgraded with a DLS (digital lightsheet). A 5× illumination objective, a 25× dipping lens together with 2.5 mm mirror caps were used to obtain a lightsheet. A stack was taken every 5 min. For imaging, glass-bottom-dishes (MatTek) were coated with 2% Agarose. Embryos were embedded in 1% low melting agarose and placed on the coating. Two notches were cut in the agarose with razorblades, so that the embryo was placed on a 2 mm wide stripe that could fit between the mirror caps. The embryo was covered with zebrafish medium, including tricaine for anaesthesia. Data from left and right eyes were used and oriented to fit the standard lateral view.

### 3.9. Tracking and visualization

Tracking of cells was performed with MTrackJ [41]. Tracks were visualized using custom-made ImageJ plugins as in [18]. The overlay images of tracks and raw data were used to produce maximum intensity projections for figure 2 and electronic supplementary material, movie S4.

### 3.10. Quantitative analysis of the length of the optic fissure

To analyse the length of the optic fissure, DAPI and anti-β-catenin-stained embryos were embedded upside down in low melting agarose. *z*-stacks with 3 µm *z*-spacing were taken. The length of the fissure was measured on an optical section below the lens, in a straight line from the embryonic retina to the surface ectoderm. The width of the OF and the eye was measured, at 50% length of the OF. The width of the neuroretina was measured in extension of the length of the OF. The length of the eye is the addition of the retina and the length of the OF. For each condition, seven eyes were measured. To determine significance, an unpaired *t*-test with Welch's correction was used, with *p* < 0.05.

### 3.11. Quantitative analysis of the contribution from the optic stalk to the neuroretina

Kaede in the optic stalk was photo-converted in a way that only the stalk and as little as possible from the optic vesicle was red. The embryo was then imaged until the flow movements were over. From every third optical section (section thickness 4 µm), the area of the optic cup was measured using the measure tool in Fiji. In the same sections, the red area was also measured and a percentage of red area in the optic cup after fissure development was calculated. This was done in three animals.

## Supplementary Material

Revision Figure 3 supplement Eckert et al 2018 upload.pdf

## Supplementary Material

Revision Figure 5 supplement 2 Eckert et al 2018 upload.pdf

## Supplementary Material

Revision Figure 5 supplement Eckert et al 2018 upload.pdf

## Supplementary Material

Revision Figure 5 supplement 3 Eckert et al 2018 upload.pdf

## Supplementary Material

Revision Figure 6 supplement 1 Eckert et al 2018 upload.pdf

## Supplementary Material

Revision Figure 6 supplement 2 Eckert et al 2018 upload.pdf

## Data accessibility

This article has no additional data.
