# Supplementary figures and images for: Morphogenesis and axis specification occur in parallel during optic cup and optic fissure formation, differentially modulated by BMP and Wnt
Source: Open Biol. 2019 Feb 13;9(2):180179. doi: 10.1098/rsob.180179 (PMC6395882; doi:10.1098/rsob.180179)

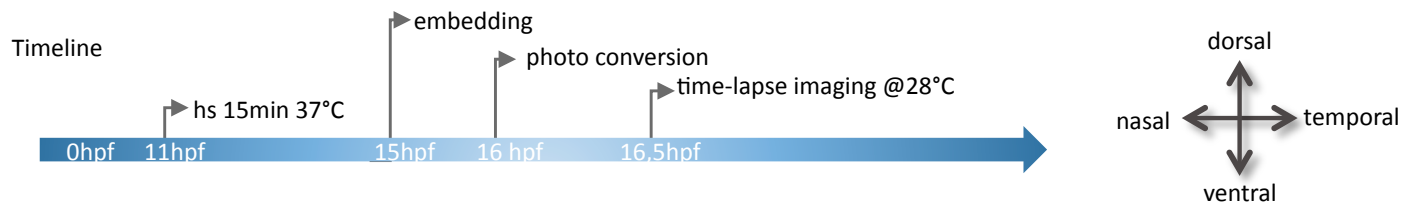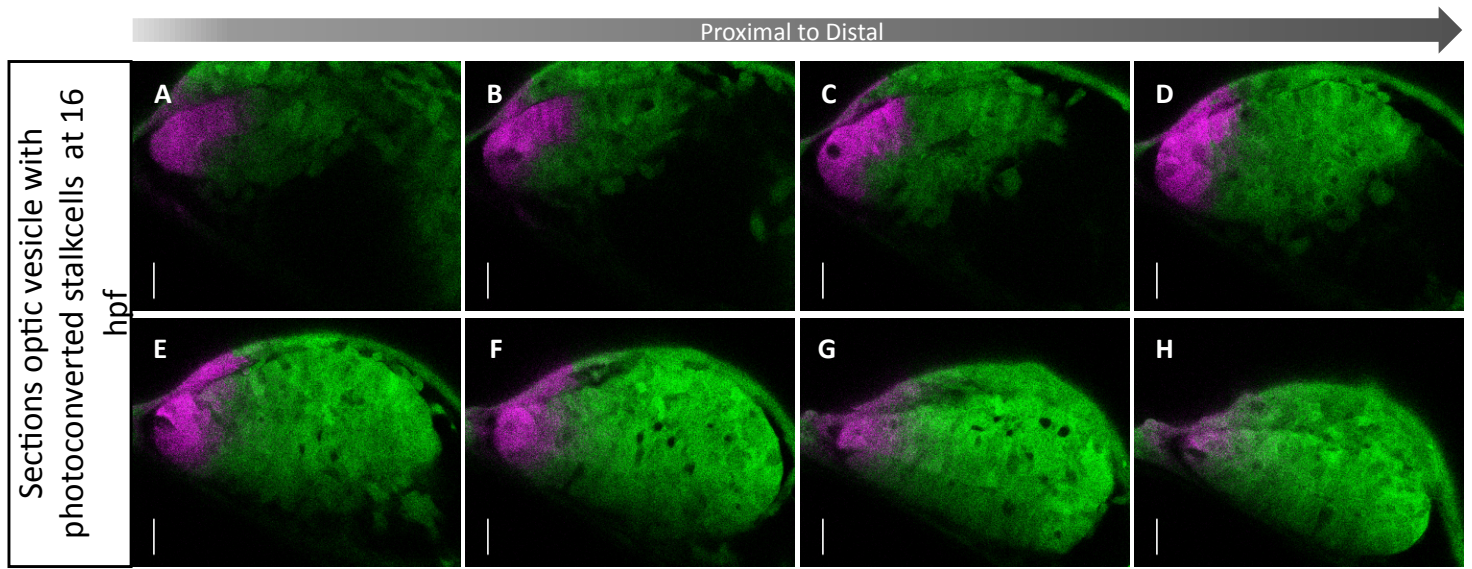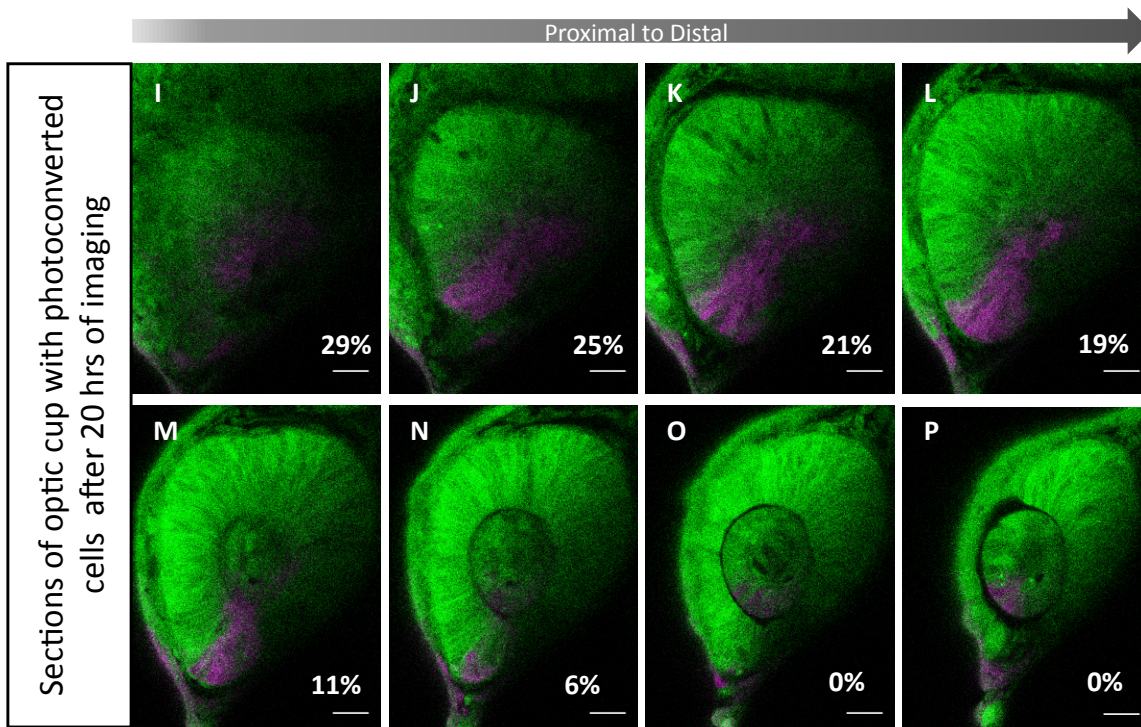

Supplement: Revision Figure 3 supplement Eckert et al 2018 upload.pdf [file rsob180179supp1.pdf]

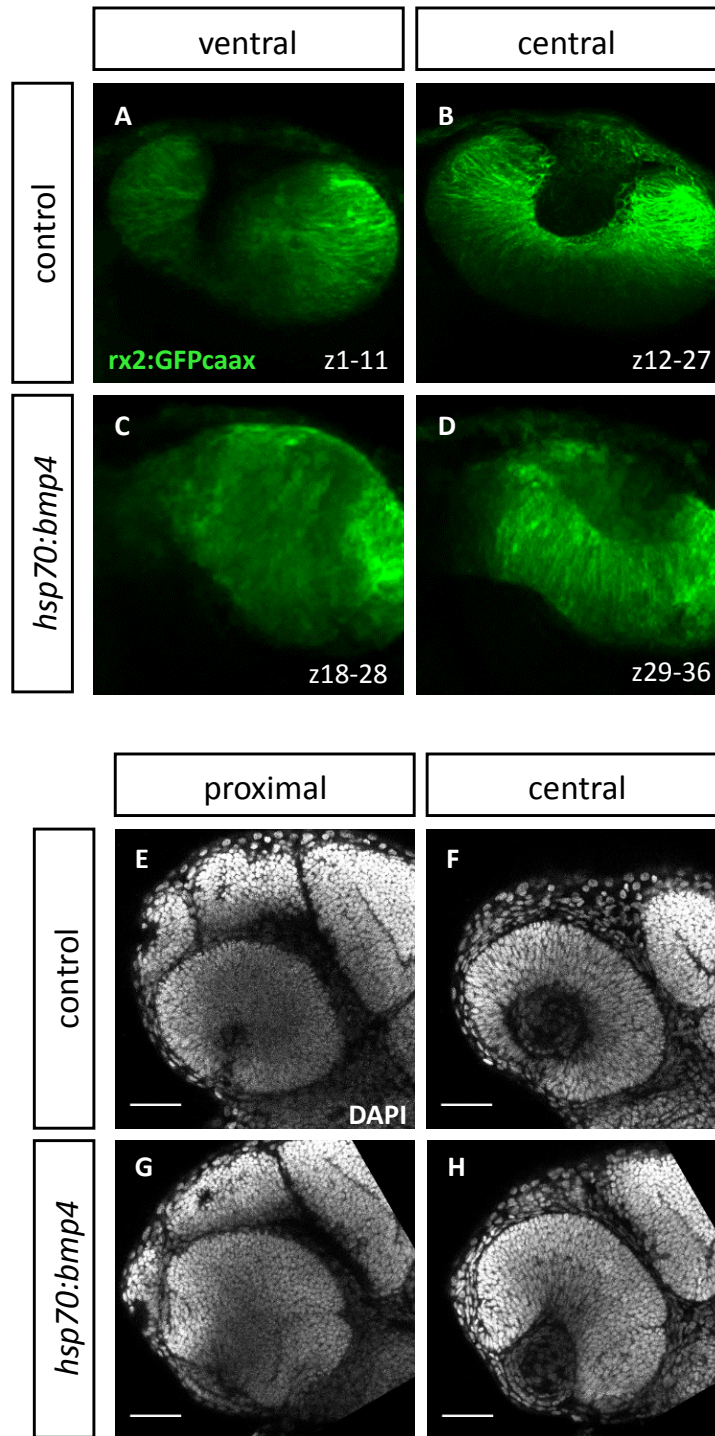

Figure 5 supplement 2, Eckert et al., 2018

Supplement: Revision Figure 5 supplement 2 Eckert et al 2018 upload.pdf [file rsob180179supp2.pdf]

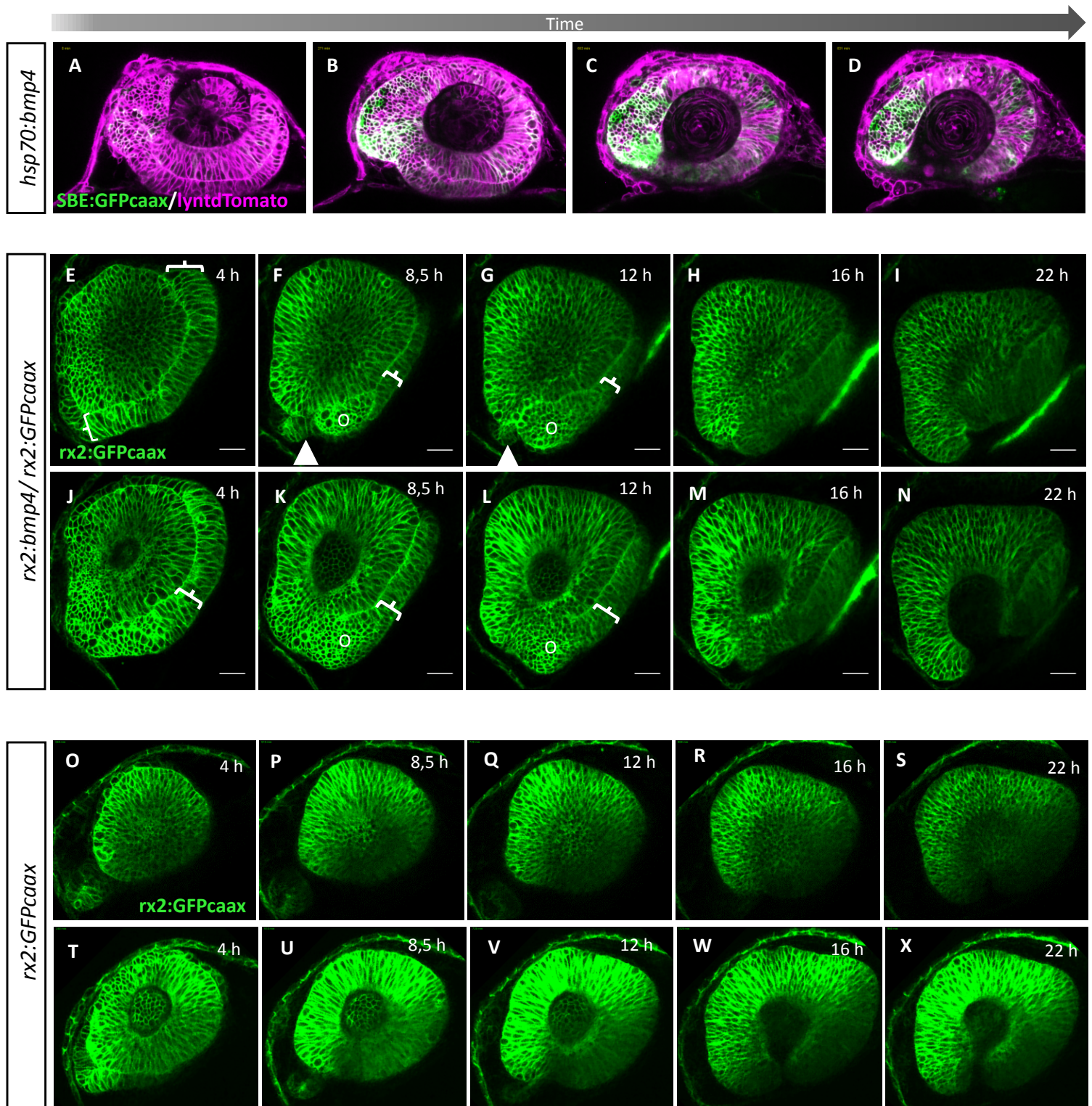

Figure 5 supplement Eckert et al., 2018

Supplement: Revision Figure 5 supplement Eckert et al 2018 upload.pdf [file rsob180179supp3.pdf]

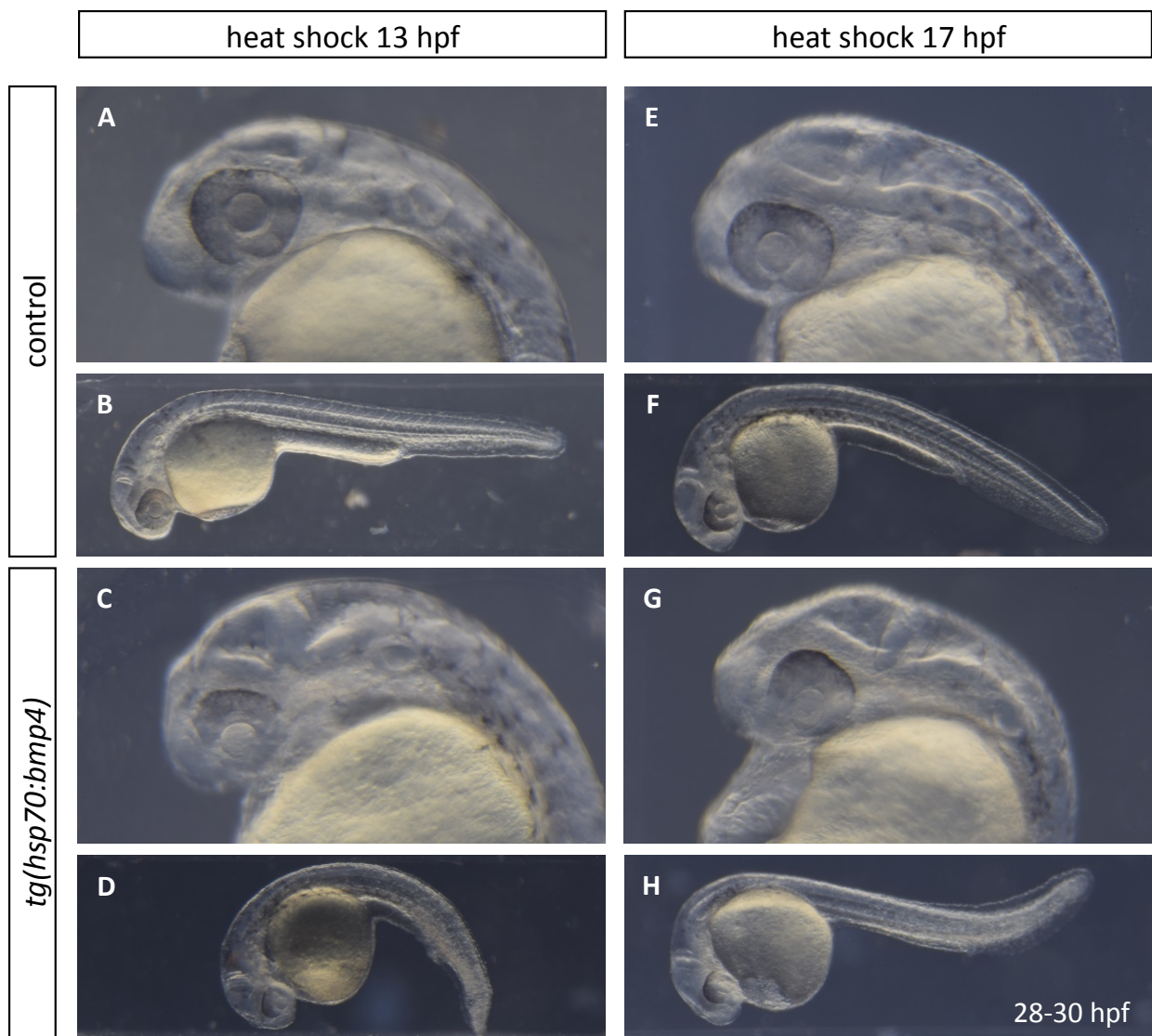

Figure 5 supplement 3, Eckert et al., 2018

Supplement: Revision Figure 5 supplement 3 Eckert et al 2018 upload.pdf [file rsob180179supp4.pdf]

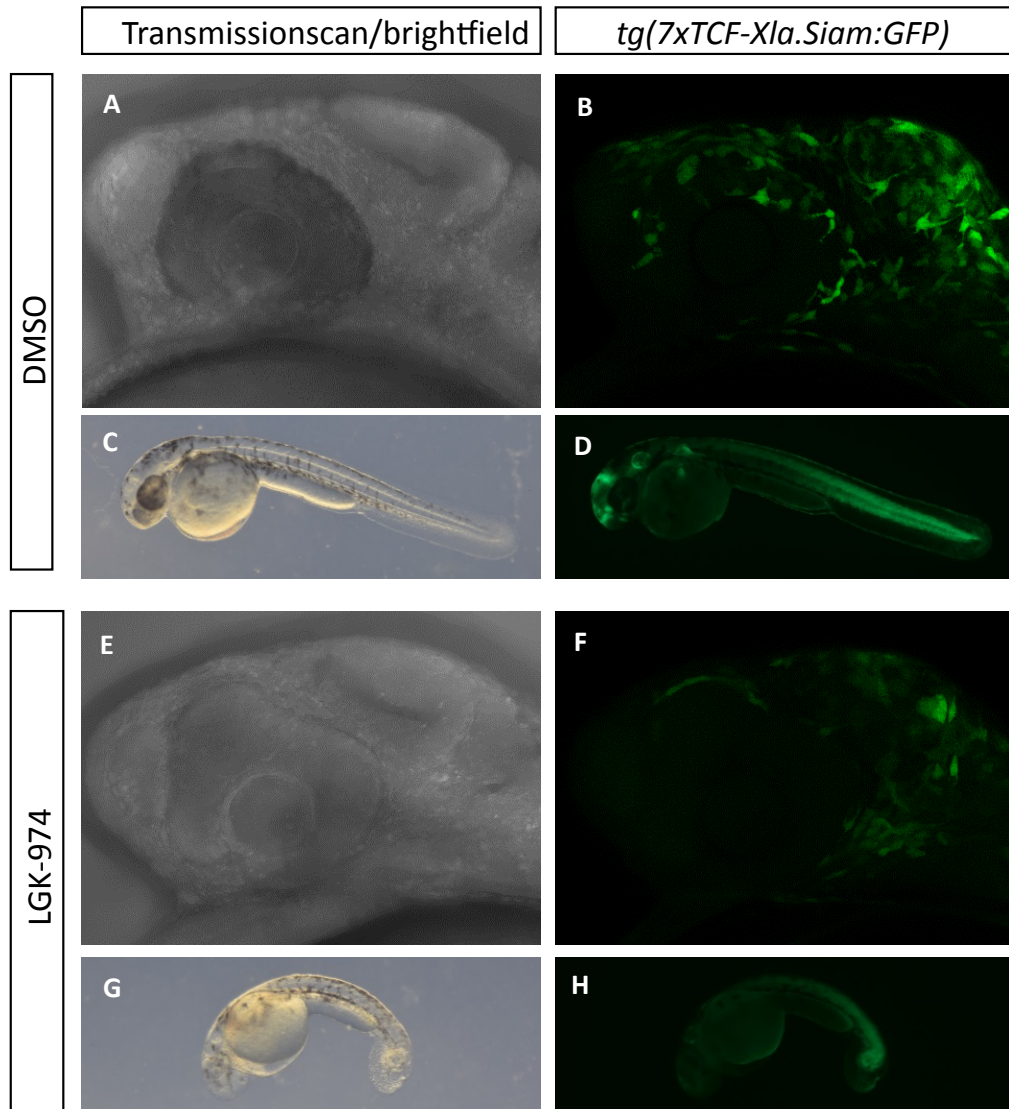

Figure 6 supplement Eckert et al., 2018

Supplement: Revision Figure 6 supplement 1 Eckert et al 2018 upload.pdf [file rsob180179supp5.pdf]

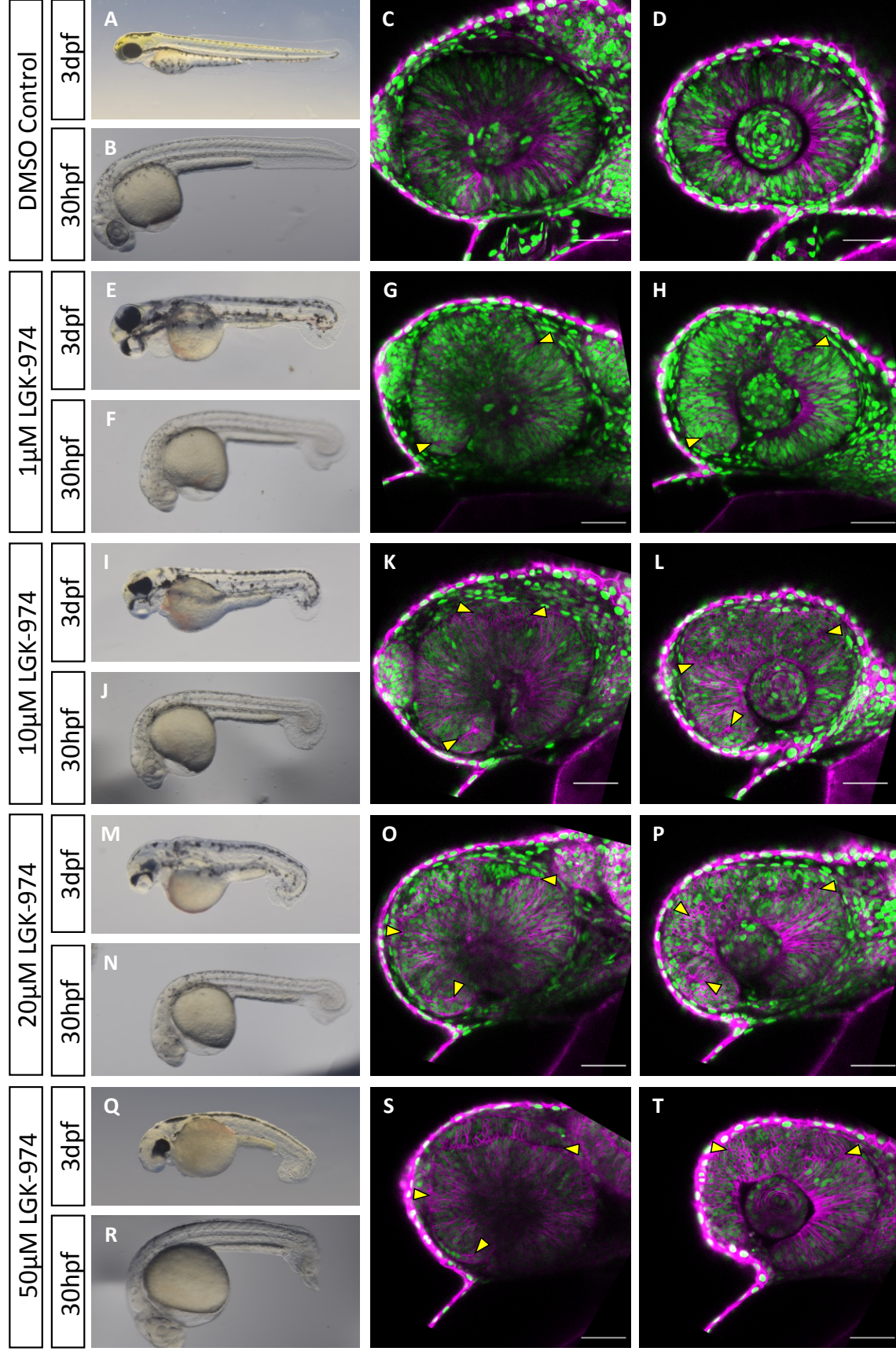

Figure 6 supplement Eckert et al., 2018

Supplement: Revision Figure 6 supplement 2 Eckert et al 2018 upload.pdf [file rsob180179supp6.pdf]
